# Vocal fold paralysis and cauda equina syndrome following spinal-epidural anesthesia: A case report

**DOI:** 10.1097/MD.0000000000024374

**Published:** 2021-01-22

**Authors:** Yuanling Xiang, Weifeng Wang, Shenfeng Jing, Zhong Zhang, Dezhang Wang

**Affiliations:** a Department of Orthopedics, Zhangqiu People's Hospital, Jinan; b Department of Orthopedic Surgery, Liaocheng People's Hospital, No.67 Dongchang West Road, Liaocheng, Shandong, PR China.

**Keywords:** cauda equina syndrome, epidural aesthesia, hallux valgus, spinal, vocal fold paralysis

## Abstract

**Rationale:**

Vocal fold paralysis and cauda equina syndrome are very rare neurologic deficits. This report describes the case of a patient who simultaneously developed both after uneventful spinal-epidural anesthesia with 0.5% hyperbaric bupivacaine.

**Patient concerns:**

We report the case of a 45-year-old female, who underwent surgery for bilateral hallux valgus developed cauda equina syndrome and unilateral vocal fold paralysis after uneventful spinal-epidural anesthesia was administered. There was no pain or paresthesia during needle placement or drug injection. Surgery was performed uneventfully.

**Diagnoses:**

Right vocal fold paralysis was diagnosed with flexible laryngoscopy.

**Interventions:**

Patient was started on the treatment with a surgery for bilateral hallux valgus, who developed cauda equina syndrome and unilateral vocal fold paralysis after uneventful spinal-epidural anesthesia was administered.

**Outcomes:**

Postoperatively, she had difficulty in urination and defecation. In addition, she developed unilateral vocal fold paralysis characterized by hoarseness, effortful voice production, and choking with liquids. Magnetic resonance imaging performed on the lumbosacral area and computed tomography of the neck, the chest, and the skull revealed entirely normal results. However, flexible laryngoscopy revealed a right vocal fold paralysis. Although cauda equina syndrome can occur due to neurotoxicity of local anesthetics, the exact etiology of vocal fold paralysis is uncertain.

**Lessons:**

The case highlights that 2 rare and serious complications of spinal-epidural anesthesia can even occur in the same patient after uneventful surgery and block performance.

## 1 Introduction

Regional anesthesia for lower limb surgeries is generally held to be inherently safe. Spinal and epidural blocks are therefore used widely, with the more recently introduced combined spinal and epidural technique gaining popularity. Serious neurologic deficits such as cauda equine syndrome and vocal fold paralysis after spinal anesthesia are relatively rare.^[1–4]^ Cauda equina syndrome is a serious neurologic disorder that is caused by damage of the lumbosacral and coccygeal nerve roots which include the ascending and descending nerve roots from L2 through the coccygeal segments. These nerves control lower limb movement (L2-S2), lower limb sensation (L2-S3), bladder control (S2-S4), external analsphincter control (S2-S4), external genitalia and perianal sensation (S2-S4), and coccygeal sensation (S4, S5, and the coccygeal nerve).^[5]^ Cauda equina syndrome is associated with varying degrees of signs and symptoms including loss of bowel and bladder function, insensate perineal areas and lower extremity muscle weakness.^[6,7]^ In addition to cauda equina syndrome, cranial nerve palsy is also a rare complication of spinal anesthesia. The most commonly affected cranial nerve is the abducent nerve because of its long intracranial course.^[8]^ Palsies of the oculomotor, facial, trigeminal, and trochlear nerves, as well as transient hypoacusis and Horner syndrome, have also been reported.^[9–13]^ Vagal neuropathy, following spinal anesthesia was first described recently.^[2–4]^ These cranial palsies are typically transient and resolve within weeks to months, but complete neurological recovery after cauda equina syndrome is unusual. The simultaneous occurrence of these 2 rare complications in the same patient has not been previously reported. This report describes the case of a patient who simultaneously developed both after uneventful spinal-epidural anesthesia with 0.5% hyperbaric bupivacaine. With early diagnosis and appropriate treatment, the patient recovered eventually.

## 2 Case presentation

A previously healthy 45-year-old female, with no preexisting neurologic deficits, was subjected to surgery because of bilateral hallux valgus. The patient's routine preoperative test results were within normal ranges. Spinal anesthesia was administered at the L3–4 interspace by using a 27-gauge Quincke point spinal needle through an 18-G Tuohy needle. Afterward, 3 ml of 0.5% hyperbaric bupivacaine was injected intrathecally in approximately 15 seconds after the clear cerebrospinal fluid (CSF) without any blood was aspirated. After bupivacaine was injected, an epidural catheter was inserted 4 cm into the epidural space in the cephalad direction via the Tuohy needle. Paraesthesia, back pain or any other signs of nerve irritation was not experienced during needle placement or drug injection. A urinary catheter was inserted before the surgery. The sensory level block was bilateral, symmetric, and caudal to T10 15 minutes after bupivacaine was administered, as assessed via the pinprick method; a complete lower extremity motor block was achieved. The patient's blood pressure decreased transiently from 140/85 mm Hg to 85/60 mm Hg; afterward, 4 mg of ephedrine was provided with hypotension resolution. The patient's arterial pressure remained normal throughout the procedure. Surgical blood loss was not significant. The 90 minutes operation was performed uneventfully. She was discharged to the ward after the operation. Ropivacaine was injected through the epidural catheter to reduce postoperative pain. After a 5 ml bolus of 0.5% ropivacaine was administered, the mixed solution composed of 0.2% ropivacaine and 4 mg/L fentanyl was infused at a basal rate of 2 ml/hour. In addition, patient-controlled epidural analgesia was maintained for almost 2 days (4 ml for 1 push, 30 minutes lockout) by using a disposable pump. The surgical procedure was conducted uneventfully with the patient in the supine position. The final sensory level achieved was T12 bilaterally by pinprick.

After surgery, the patient complained of mild headache. Thereafter, neck stiffness and pain, photophobia, nausea and vomiting also occurred. The next day of the operation, she felt a sudden weakness in her left leg but still able to walk with an assistive device. Then, the weakness increased as well as the right leg. Unexpectedly, she complained of numbness in the gluteal and perianal region. The epidural catheter was removed immediately. According to the International Standards for Neurological Classification of Spinal Cord Injury (ISNCSCI), which developed by the American Spinal Injury Association (ASIA) and endorsed by the International Spinal Cord Society, is a well-accepted classification of neurologic deficit after Spinal Cord Injury (SCI),^[14,15]^ the examination used dermatomal light touch and pin prick sensation and motor strength of selected muscle groups of the lower extremities to determine the neurologic level of the injury. In the patient, physical examination at that time revealed bilateral lower extremity weakness with 3/5 for left hip flexors and knee extensors; 2/5 for left ankle dorsiflexors and plantar flexors, 1/5 for left extensor hallucis longus; 4/5 for the right hip flexors and knee extensors; 3/5 for right ankle dorsiflexors and plantar flexors, 2/5 for right extensor hallucis longus. Motor scores of the 2 lower limbs were 27 points. Neurological examination documented lower extremity and perianal hypoesthesia and lower extremity hyporeflexia. We defined lower extremity pin prick and light touch scores as the sum of L2 to S5 dermatomes bilaterally (maximum score of 32 points). Pinprick sensation of the left L4-S1 segment, right L4-L5 segment and perianal area in the S3–5 segment was diminished. Pin prick and light touch scores of the left and right lower limbs were 22 and 24 points respectively. The Achilles and patellar reflexes were obviously reduced, and the anal wink reflex was absent in this patient. The anal sphincter tone was decreased. The abdominal reflexes and strength of abdominal wall muscles were bilaterally normal. Three days after surgery, the symptom of headache faded away gradually. The patient was unable to micturate when the urinary catheter was removed on the third postoperative day; as a result, urinary retention (on the third day) and rectal incontinence (on the fourth day) occurred. The bladder was recatheterized because of the urinary retention. Seven days after surgery, Magnetic resonance imaging (MRI) was performed on the lumbosacral area to obtain further information. The MRI revealed no tumor, hematoma, abscess, infarction, spinal stenosis, or cauda equina nerve root compression. Based on the patient's symptoms and MRI findings, cauda equina syndrome was diagnosed. In addition to cauda equina syndrome, she noted onset of hoarseness, effortful voice production and chocking with liquids on postoperative day 5; she believed that her voice had been normal before and immediately after surgery. She subsequently underwent flexible laryngoscopy, which revealed a right vocal fold paralysis. Computed tomography (CT) of the neck and chest was performed 10 days after surgery, which did not reveal any lesions along the recurrent laryngeal nerve. The brain MRI findings showed: diffuse dural enhancement, downward displacement of the brainstem, and diminution of cerebral ventricles, which are typical features of intracranial hypotension (ICH). Stroboscopy performed 15 days after surgery confirmed the finding of an immobile right vocal fold.

The patient was managed conservatively on heavy doses of steroids:^[16]^ methylprednisolone 500 mg intravenously guttae daily for 5 days, then reduced to 80 mg intravenously guttae daily for 10 days. She also underwent a rehabilitation program including traditional Chinese medicine and acupuncture 3 days per week, which aimed to normalize movement patterns. Physical therapy for cauda equine syndrome included static and dynamic control of position, neurophysiologic exercises, progressive ambulation, balance skills, and activities of daily living. Clean intermittent self-catheterization 3 times a day was started for neurogenic bladder. Vocal therapy in unilateral vocal fold paralysis consisted of electrotherapy, hard glottal attacks and pushing exercises, half-swallow boom, accent method, lip and tongue trills. After 2 months, her lower extremity motor strength significantly improved and the sensation of the 2 lower limbs recovered gradually except hypoesthesia in the S3–5 segment. Motor scores of the 2 lower limbs were 45 points; pin prick and light touch scores of the left and right lower limbs were 26 and 28 points, respectively. At the 3-month follow-up, the patient had regained 5/5 strength in all motor groups, could walk normally without any assistive devices and perianal sensation somehow returned. At the 11-month follow-up, the patient recovered from urinary retention and fecal incontinence. In addition, a laryngeal electromyography, performed at 3 months following surgery, showed evidence of denervation of the right thyroarytenoid (innervated by the recurrent laryngeal nerve) and cricothyroid muscle (innervated by the superior laryngeal nerve), consistent with a right vagal neuropathy; at 7-month follow-up, the patient reported that her voice quality improved to near normal and only mild residual paresis was noted on stroboscopy.

## 3 Discussion and conclusions

The patient developed cauda equina syndrome characterized by urinary retention and fecal incontinence, perianal sensory loss, and lower extremity weakness after uneventful routine spinal-epidural anesthesia. She simultaneously developed a right vocal fold paralysis. Although these major complications rarely occur after spinal-epidural anesthesia, the described patient developed both.

Various possible causes achieved vocal fold paralysis. It may be infectious, neoplastic, or idiopathic in origin. There was no evidence of infection in this case. The CT ruled out the possibility of neoplasm. At first glance, this case of vocal fold paralysis may be classified as idiopathic because spinal anesthesia is generally not considered in the differential diagnosis of vocal fold paralysis. However, a causal relationship may be considered according to the documented occurrence of upper cranial neuropathy as a result of spinal anesthesia, especially in the absence of other important contributing factors. The onset of symptoms is consistent with previously reported cases of cranial neuropathy following spinal anesthesia. Guardiani et al^[2]^ reported 4 cases of vocal fold paralysis after spinal anesthesia. Cranial nerve palsy following subarachnoid block or unintentional dural puncture has been attributed to CSF depletion and ICH, which appears to be the cause of vocal fold palsy.^[13]^ ICH is a clinical syndrome characterized by postural headache and low CSF opening pressure. Symptoms may include nausea, vomiting, nuchal pain, dizziness, tinnitus, nystagmus, and blurred vision.^[3]^ The MRI findings of ICH are diffuse pachymeningeal enhancement, subdural effusion, engorgement of venous sinuses, pituitary enlargement, and herniation of the cerebellar tonsils.^[17]^ It has been explained on the basis of CSF loss causing sagging of the brain and stretching of the nerve.^[18]^ The brain descends caudally to a vertical position, which may harm the cranial nerves that anchor the brain to the skull. In the described patient, laryngeal electromyography suggested a vagal lesion, in light of the evidence of denervation of both the cricothyroid and thyroarytenoid muscle, rather than an isolated neuropathy of the recurrent laryngeal nerve; this supports the hypothesis of traction on the vagal nerve rather than an idiopathic palsy, the majority of which are recurrent nerve phenomena. Therefore, the decrease in intracranial CSF pressure may have contributed to the presentation, which may induce stretching of the vagal nerve bilaterally. In this case, only the right vocal fold was immobile, therefore, the vagal nerve could be stretched with 1 side more affected than the other. Hypotension during surgery may also serve as a contributing factor to this complication because vascular perfusion of nerve fibers might be compromised. Thus the explanation for vocal fold paralysis is uncertain and consequently speculative.

Cauda equina syndrome is a rare but an extremely distressing complication of spinal-epidural anesthesia.^[19–21]^ Most neurological complications are caused by epidural hematoma, abscess, catheter trauma, infection, and anesthetic toxicity. In the patient, the MRI revealed no hematoma, abscess, infarction, spinal stenosis, or lumbar disc herniation. Direct nerve damage may contribute to the onset of neurological symptoms; disposable needle tips are very sharp, nerve fibers can easily be injured by them. Most neural injuries are associated with either paraesthesia or pain upon injection.^[22]^ However, direct damage to the spinal cord via dural puncture is unlikely the cause of the condition observed in the patient because neurologic pain or paraesthesia was not experienced during needle insertion.^[23,24]^ Spinal cord compression caused by an expanding hematoma was excluded through MRI. Infection was not observed because the patient did not manifest clinical signs or laboratory abnormality corresponding to infection. Dural blood flow is significantly reduced in animals once epinephrine is added to local anesthetic solutions.^[25]^ However, spinal cord ischemia was unlikely observed in this case because epinephrine that can induce vascular contraction was not administered, the patient did not suffer from microvascular disease and was hemodynamically stable perioperatively.^[26]^ Rigler et al^[27]^ postulated that a combination of trauma, maldistribution, and relatively high dose of local anesthetic resulted in neurotoxic injury. Most cauda equina syndrome cases following spinal anesthesia are reportedly associated with toxicity of local anesthetics, especially lidocaine. Limited cephalad extension of sensory blocks suggests restricted diffuse of lidocaine in the cerebrospinal fluid and is likely to induce anesthetic neurotoxicity. Several cases of cauda equina syndrome following spinal anesthesia with bupivacaine have been reported. Kato et al^[28]^ reported a cauda equina syndrome case following spinal anesthesia with bupivacaine and epidural anesthesia with ropivacaine, which took 10 months to recover. Similarly, Chabbou et al^[29]^ reported a cauda equina syndrome case following spinal anesthesia with 0.5% hyperbaric bupivacaine without any causative factors in the genesis of the syndrome. Therefore, based on the patient's symptoms and MRI findings, the outcome was probably caused by the neurotoxicity of bupivacaine alone or in combination with ropivacaine. In contrast to other patients, the patient in this report recovered almost completely. This may be the result of the early detection and therapy of the neurologic complication.

In summary, the case highlights that 2 rare and serious complications of spinal-epidural anesthesia can even occur in the same patient after uneventful surgery and block performance. The neurotoxicity of local anesthetics, even with normal dose, is suggested as the most likely cause of cauda equina syndrome, and ICH caused by leakage of CSF serves as a potential factor in causing vocal fold paralysis. Therefore, detailed physical examination, and monitoring recovery of sensory and motor functions following spinal-epidural anesthesia are significant for early detection and therapy of neurologic complications. Notably, practitioners should detect and deal with these rare types of neurologic complications as early as possible, the patient should be investigated thoroughly, and appropriate treatment and rehabilitation program should be initiated promptly. In this patient, early diagnosis and intervention of vocal fold paralysis is crucial to manage it; otherwise, permanent injuries may likely occur.

## Author contributions

**Conceptualization:** Yuanling Xiang, Weifeng Wang, Shenfeng Jing, Zhong Zhang, Dezhang Wang.

**Data curation:** Yuanling Xiang, Weifeng Wang, Shenfeng Jing, Zhong Zhang, Dezhang Wang.

**Formal analysis:** Yuanling Xiang, Weifeng Wang, Shenfeng Jing, Zhong Zhang, Dezhang Wang.

**Investigation:** Yuanling Xiang, Weifeng Wang, Shenfeng Jing, Zhong Zhang, Dezhang Wang.

**Methodology:** Yuanling Xiang, Weifeng Wang, Shenfeng Jing, Zhong Zhang, Dezhang Wang.

**Project administration:** Yuanling Xiang, Weifeng Wang, Shenfeng Jing, Zhong Zhang, Dezhang Wang.

**Resources:** Yuanling Xiang, Weifeng Wang, Shenfeng Jing, Zhong Zhang, Dezhang Wang.

**Software:** Yuanling Xiang, Weifeng Wang, Shenfeng Jing, Zhong Zhang, Dezhang Wang.

**Supervision:** Weifeng Wang, Shenfeng Jing, Zhong Zhang, Dezhang Wang.

**Validation:** Yuanling Xiang, Weifeng Wang, Shenfeng Jing, Zhong Zhang, Dezhang Wang.

**Visualization:** Yuanling Xiang, Weifeng Wang, Shenfeng Jing, Zhong Zhang, Dezhang Wang.

**Writing – original draft:** Yuanling Xiang, Weifeng Wang, Shenfeng Jing, Zhong Zhang, Dezhang Wang.

**Writing – review & editing:** Yuanling Xiang, Weifeng Wang, Shenfeng Jing, Zhong Zhang, Dezhang Wang.
